# An Unusual Case of Chest Pain Presenting to the Cardiology Clinic

**DOI:** 10.7759/cureus.71622

**Published:** 2024-10-16

**Authors:** Arjun Khunger, Mohammad Karim, Sawyer Bawek, Ankita Kapoor, Nikhil Agrawal

**Affiliations:** 1 Department of Hospital Medicine, PeaceHealth Sacred Heart Medical Center, Springfield, USA; 2 Department of Medicine, Division of Cardiology, University of Pittsburgh Medical Center Hamot, Hamot, USA; 3 Internal Medicine, University at Buffalo, Buffalo, USA; 4 Hematology and Oncology, Roswell Park Comprehensive Cancer Center/University at Buffalo, Buffalo, USA; 5 Department of Medicine, Division of Cardiology, University of Pittsburgh Medical Center Chautauqua, Jamestown, USA

**Keywords:** cardiac ischemia, chest pain in the young, clinical cardiology, exertional chest pain, mitral valve disease, mri cardiac, pectus excavatum, primary care clinic, unusal causes of persistent chest pain

## Abstract

We present the case of a 45-year-old physically active female who presented to the cardiology clinic with subacute chest pain. Despite an extensive diagnostic cardiac workup revealing no significant findings, her chest pain persisted. The patient was finally referred to thoracic surgery for the management of severe pectus excavatum deformity, and her symptoms improved following surgical correction of the condition. Our case report details a symptomatic case of pectus excavatum, emphasizing the importance of recognizing that this condition can be associated with clinical symptoms.

## Introduction

Chest pain is a frequent chief complaint with a broad differential diagnosis that includes several life-threatening conditions. Initial diagnostic assessment should focus on excluding serious pathologies before considering more benign etiologies. In many cases, benign causes are overlooked when ischemic workup is negative. In these instances, further investigation into less apparent causes is warranted, as chest pain can lead to significant morbidity and anxiety for patients.

Pectus excavatum is the most common congenital chest wall skeletal deformity. It is reported to occur in approximately 1 in 1,000 live births [1]. It is characterized by a malformation of the anterior chest wall where the lower portion of the sternum is displaced posteriorly. This concave chest deformity can result in compression and displacement of the thoracic structures. In one study of 50 adults with pectus excavatum, about 42% reported shortness of breath and 68% had associated chest pain [2,3]. As a result, multiple surgical techniques have been developed that have shown successful resolution of symptoms in affected patients [4-6]. Here, we report a patient with severe pectus excavatum requiring surgical correction for symptom relief.

## Case presentation

A 45-year old Caucasian female presented to the cardiology office with a subacute onset of substernal chest pain for the past two to three weeks. This pain was predominantly localized to the right side of the chest. It was exacerbated by physical exertion and emotional stress, with no identifiable relieving factors. She also complained of intermittent palpitations for the last two to three weeks. Additionally, she reported mild dyspnea on exertion during these episodes but no syncope, fever, cough, and other relevant symptoms.

Her medical history was significant for hyperlipidemia and mixed connective tissue disorder with rheumatoid arthritis/Sjogren’s syndrome/lupus. She was a former long-term tobacco user (10-pack-year history) but had transitioned to active vaping. The patient denied significant alcohol or illicit drug use. She was not on any medicines before this visit except as needed Tylenol which provided no relief from her chest pain. There was no significant family history of ischemic heart disease.

In the clinic visit, her vitals included a heart rate of 72 beats per minute, blood pressure of 116/88 mmHg, respiratory rate of 19 breaths per minute, and temperature of 98.4°F. On the physical examination, cardiac auscultation revealed a regular rate and rhythm without murmurs or added sounds. No jugular venous distension was noted. A significant pectus excavatum deformity was noted on examination.

**Figure 1:**
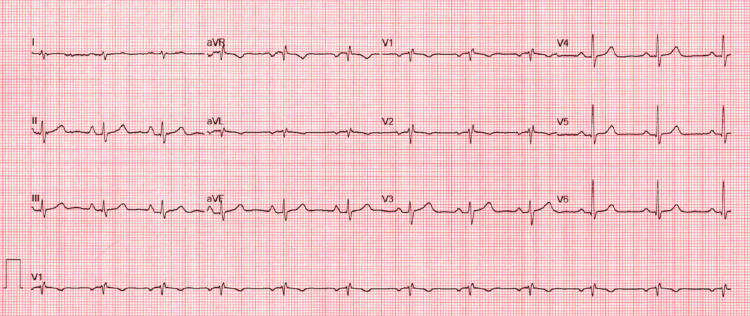
Electrocardiogram showing sinus rhythm with an indeterminate axis.

The initial electrocardiogram (Figure 1) showed normal sinus rhythm with an indeterminate axis. The patient was also placed on a cardiac monitor given her complaints of palpitations and was found to have 1% premature ventricular contractions (PVCs) over a 14-day cardiac event monitoring period but an otherwise unremarkable study. Cardiac biomarkers including troponin-I and B-type natriuretic peptide level were normal at <0.01 ng/mL and 16 pg/mL, respectively normal (Table 1). Chest X-ray showed no acute cardiopulmonary disease and was otherwise unremarkable.

**Table 1:** Cardiac biomarkers including the normal reference values.

Laboratory parameters	Value	Normal value
Troponin-I	<0.01 ng/mL	Between 0 and 0.04 ng/mL
B-type natriuretic peptide (BNP)	16 pg/mL	Less than 100 pg/mL

She underwent a transthoracic echocardiogram in the office that showed a left ventricular ejection fraction (LVEF) of 45-50% with regional wall motion abnormalities, including severe hypokinesis of the apical septum and left ventricular (LV) apex. Given the echocardiogram findings, there was concern of Takotsubo stress-induced cardiomyopathy versus obstructive coronary heart disease of the left anterior descending artery. She underwent cardiac catheterization that revealed normal coronaries without any evidence of obstruction.

**Figure 2:**
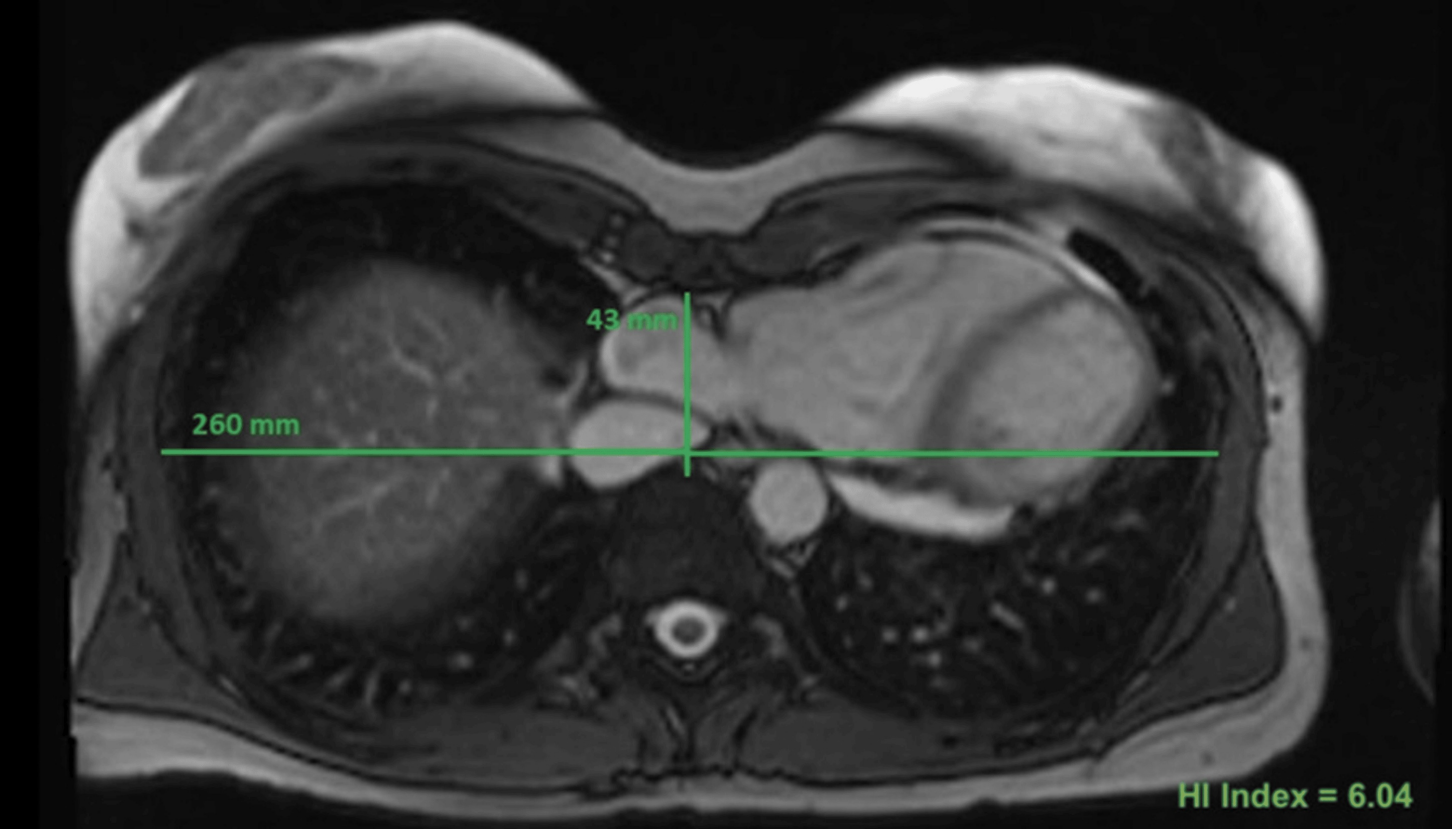
Cardiac magnetic resonance imaging depicting cardiac displacement and compression of right-sided cardiac chambers and a Haller index of 6.

She was started on Tylenol 650 mg three times daily to assess whether her chest pain had a musculoskeletal origin. Additionally, she was prescribed metoprolol succinate 12.5 mg XL nightly for the management of her palpitations. A subsequent cardiac magnetic resonance imaging (MRI) revealed an LV function of 52% with a normal right ventricular (RV) EF of 51%. The imaging showed mitral annular dysjunction with a 4 mm separation between the mitral annulus and myocardium during systole. Systolic guarding of the basal inferolateral wall was observed. There was no significant mitral regurgitation, although flattening of the mitral valve along the annular plane during systole was noted, without prolapse. There was no evidence of late gadolinium enhancement, indicating the absence of scar or fibrosis. No shunt was detected, with a Qp/Qs ratio of 1.04. Extracardiac findings included severe pectus excavatum with a Haller index of 6.04 (Figure 2). Genetic testing to rule out Marfan syndrome returned negative, leading to the conclusion that the patient has an isolated case of severe pectus excavatum deformity.

The patient was seen again in the clinic following the completion of her diagnostic workup and her symptoms had shown only slight improvement with the prescribed medications. The patient was subsequently referred to a pediatric thoracic surgeon for evaluation of the surgical indications for pectus excavatum repair. The patient underwent the Nuss procedure and recovered well. At a follow-up visit in the cardiology clinic a few months later, an echocardiogram was performed which showed low normal LVEF of 50% with improved regional wall motion abnormalities with mild hypokinesis of the apical septum and LV apex. Moreover, the patient no longer reported chest pain and her symptoms of exertional dyspnea, fatigue, and palpitations had fully resolved. The patient expressed satisfaction with her recovery, expressing that she will be able to resume her physical activity.

## Discussion

Pectus excavatum is generally considered a benign condition, often presenting with minimal symptoms or requiring no treatment. However, studies have demonstrated that severe cases may have clinical symptoms, including exercise intolerance, chest pain, shortness of breath, and palpitations [2,3]. Symptoms tend to peak in adolescence. The severity of pectus excavatum can be calculated by the pectus severity index (PSI) or Haller index that is derived from dividing the transverse diameter of the chest by the anteroposterior diameter as measured by imaging. Normal values of the Haller index are less than 2.5 [7]. A severe Haller index in pectus excavatum is generally considered to be greater than 3.25. This threshold is widely accepted as an indication for surgical correction due to the associated cardiopulmonary implications [8,9].

In this patient, given the negative cardiac workup, her chest pain and exertional shortness of breath were most likely attributable to severe pectus excavatum. She was likely experiencing cardiopulmonary limitations due to significant restriction within the chest cavity, as indicated by a severe Haller index of 5.1. Following surgical correction, her symptoms improved dramatically, and she no longer reported experiencing these issues. Similar studies have demonstrated that symptoms are significantly reduced or completely resolved after the correction of pectus excavatum [3].

Apart from chest pain, patients with pectus excavatum may have higher incidence of valvular disorders [3] , arrhythmias [10], or RV dysfunction [11]. Cardiac compression can potentially lead to distortion or compression of the mitral annulus, which may contribute to subsequent mitral annular dysfunction. It has been suggested that mitral annular dysfunction may serve as a recurrent focus for arrhythmias and PVCs, which can also contribute to the patient’s chest pain and palpitations. In severe cases, mitral valve prolapse or mitral regurgitation have been observed [3]. Fortunately, our patient did not have any significant mitral valve prolapse or mitral regurgitation. The question arises as to why the patient experienced subacute chest pain despite having a congenital deformity such as pectus excavatum. We suspect that emotional stress, combined with episodes of PVCs, acutely triggered the chest pain that required her to seek medical evaluation. It is possible that the patient had previously exhibited subclinical symptoms, which went unnoticed due to her young age and high level of physical activity.

## Conclusions

The present case report details a case of symptomatic pectus excavatum, underscoring the importance of recognizing that pectus excavatum is not always an incidental finding. This information is relevant for both primary care physicians and cardiologists who should consider pectus excavatum as a potential etiology for symptoms, especially in the absence of other satisfactory explanations.
